# Supplementary material for: Evolution of the Probe-Based Loop-Mediated Isothermal Amplification (LAMP) Assays in Pathogen Detection
Source: Diagnostics (Basel). 2023 Apr 24;13(9):1530. doi: 10.3390/diagnostics13091530 (PMC10177487; doi:10.3390/diagnostics13091530)
Supplement: Supplementary file 1 [file diagnostics-13-01530-s001.zip › diagnostics-2297425-supplementary.pdf]

Supplementary Table S1. Information list of portable fluorescence analyzers.

Country	Manufacturer	Model number
United States	Tetracore	T-COR8
Britain	IT-IS	My go mini/pro
South Korea	Ahram Biosystems	Palm
Taiwan, China	Blue-Ray	Mini Turbo Q
Taiwan, China	GeneReach	POCKIT
Beijing, China	Annoron	ANY246
Beijing, China	Genpoct	GPT-L08
Jiangsu, China	Tianlong	Gentier mini/mini+
Zhejiang, China	Bio-Gener	H1600/H8800 super mini
Zhejiang, China	COYOTE	Mini8 Plus
Zhejiang, China	Molecular	Q160/Q320
Zhejiang, China	Bioer technology	FQD-16A
Zhejiang, China	Allsheng	Esan-Gene
